# Serum progesterone screening for frozen embryo transfer: present and future perspectives

**DOI:** 10.5935/1518-0557.20190013

**Published:** 2019

**Authors:** Buenaventura Coroleu, Sofia Gaggiotti-Marre

**Affiliations:** 1 Dexeus Mujer. Departament of Obstetrics, Gynecology and Reproduction, University Hospital Dexeus, Barcelona, Spain

Artificial reproductive technologies (ART) have rapidly evolved over the past decades in order to improve the reproductive outcomes of infertile couples, including great advances in the cryopreservation process (Rienzi *et al.*, 2017), as well as in the field of Preimplantational Genetic Testing for Aneuploidies (PGT-A) (Coates *et al.*, 2017). Although endometrial preparation for frozen embryo transfer (FET) can be accomplished in a natural, natural-modified or an artificial cycle, artificial endometrial preparation allows an easier programming of the embryo transfer (ET), and thus is frequently the preferred strategy by clinicians.

The timing of endometrial receptivity is brief and failure of the endometrium to achieve a receptive state is thought to be a key factor for infertility and a major challenge for most reproductive medical clinicians. Endometrial receptivity seems to be driven by time of progesterone (P) exposure, only after sufficient exposure to estrogen and therefore P is absolutely necessary for embryo implantation and the maintenance of pregnancy (Gellersen & Brosens, 2014).

The impact of serum P in FET cycles has been widely studied; with evidence suggesting that luteal phase P supplementation improves live birth rates. It could be speculated that a certain serum P value should be attained for an adequate immunological environment to allow implantation to occur and reduce pregnancy loss. Many attempts have been made to find whether there is an optimal serum P value around the time of ET and on the day of pregnancy test, as well as whether there is an ideal route for P supplementation. In this regard, a recent study by our group indicates that low serum P value (<10.64ng/mL) the day before FET of euploid embryos is associated to higher miscarriage rates and lower live birth rates (Gaggiotti-Marre *et al.*, 2018). One of the main differences between the aforementioned study and previous ones (Labarta *et al.*, 2017) is that it focuses only on genetically-tested blastocysts, and that serum P levels are measured one day before ET and not on the same day.

The clinical implications of these findings suggest that an intervention is still possible at this stage, when the embryo has not yet been transferred into the uterus. Similarly, a recent study (Alsbjerg *et al.*, 2018) found that serum P value <11 ng/mL the day of pregnancy test was related to worse pregnancy outcomes. These studies raise the clinical question of whether there is still room for improvement in terms of luteal phase support: is it possible to increase serum P before ET? If so, how can it be done and what are the implications? Does the time of serum P measurement affect its result? Is it useful to measure serum P levels on the day of the pregnancy test? If so, what is the impact and how could it be overcome? In this regard, future studies should be performed aiming at detecting and treating patients with low serum P value the day before ET or the day of the pregnancy test, as an attempt to improve their pregnancy outcomes.

For many years, ART have focused on improving follicular recruitment, oocyte yield after pick up and subsequently, obtaining the best possible embryo, with little emphasis on luteal phase support and its repercussions on the final outcome. Yet, evidence suggests there is an unquestionable role of P for pregnancy achievement and maintenance, once again providing a new opportunity for greater advances in the field of reproductive medicine.
